# From discovery to surgery: A case report on an extremely rare coronaro-cava fistula

**DOI:** 10.1016/j.heliyon.2024.e40669

**Published:** 2024-11-23

**Authors:** F. Bouriche, S. Yvorra, V. Gariboldi

**Affiliations:** a Centre Hospitalier de Martigues, France; b CHU Timone, France

**Keywords:** Fistula, Cardiac imaging, Cardiac surgery

## Abstract

Coronary fistula are rare and of various anatomic or clinical presentations. We report the case of a patient presenting a coronaro-cava fistula from its discovery to the surgery.

## 1 Introduction

Coronary fistulas are relatively rare anomalies of connection between a coronary artery and a heart chamber or a thoracic vessel, the first anatomical description of which dates back to 1841 in Austria [1]. The impact is, of course, underestimated. The literature reports 0.002 % of fistulas among the 0.2–1.2 % of known coronary abnormalities, the rarest being the coronarocaval fistula [1,2]; 0.1–0.5 % of diagnostic coronarographies reveal the presence of a coronary fistula [3]. 90 % of fistulas are unique and in 75 % of cases they are asymptomatic [1]. We will present here the very rare case of a coronaro-cava fistula.

## 2 Case presentation

The story begins in 2011, when Mr C, then 56 years old, consulted a cardiologist.

This patient, a former hospital public service retiree (senior health executive), presents as notable history: treated hypertension, former smoker 20 pack year history, chronic alcoholism in process of weaning, obesity evolving since 2007 (BMI>30), coronary heredity. He complains of several dizziness, such as a feeling of impending death with some sweating. Clinical examination and ECG are without notable abnormality.

**Fig. 1:**
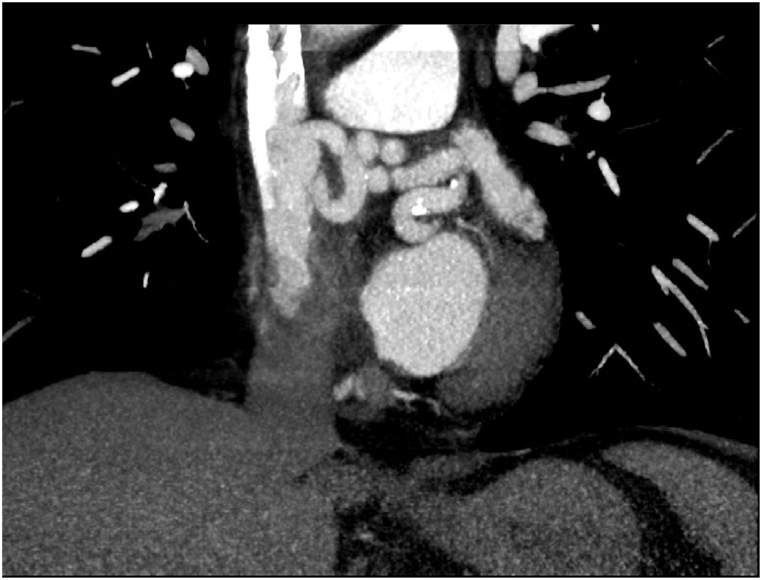
Sagittal section CT with sinuous fistula circumflex artery and superior vena cava.

**Fig. 2:**
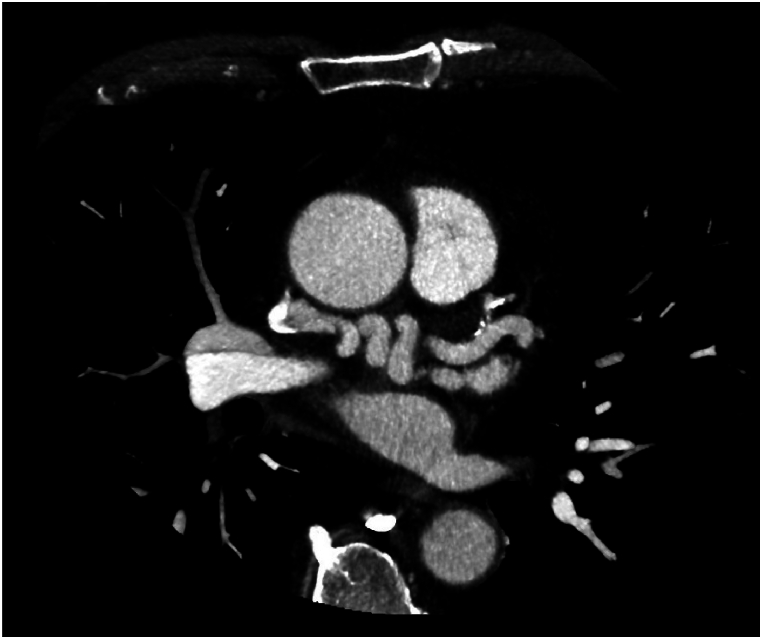
Axial section CT with sinuous fistula circumflex artery and superior vena cava.

Paraclinical examinations are undertaken, the chronology of which is as follows:

A cardiac CT which shows a large left main coronary artery at 26mm supplying a sinuous fistula with a medium diameter between 7 and 8 mm, between the Circumflex artery and the Superior Vena Cava (Fig. 1, Fig. 2), a myocardial scintigraphy which does not find myocardial ischemia and no evidence supporting ischemia steal, a transthoracic echocardiography which finds a retro-aortic laminar flow with diphasic diastolic Doppler flow compatible with a probable fistula. However, heart chambers are not dilated, and no hemodynamic shunt is found.

The case makes several practitioners think in a multidisciplinary way in front of the rarity of the pathology: attending cardiologist, radiologist, coronarographist and cardiac surgeon. From 2011 to 2020, the patient is monitored annually (transthoracic echocardiography and cardiac CT) and no longer presents any symptoms of the cardiovascular lineage, on cardiac CT fistula remains stable and no dilatation of heart chambers is found. There is no hemodynamic impact nor echocardiography abnormalities.

**Fig. 3:**
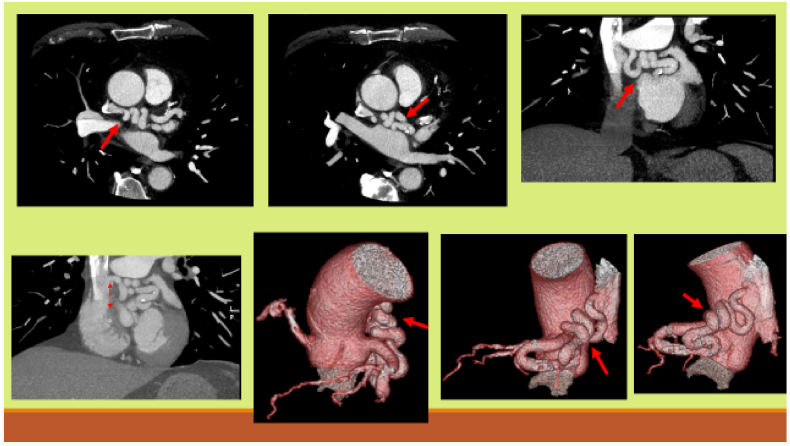
Several CT sections of the fistula.

In October 2020, on the annual cardiac CT scan, as usual the coronaro-cava fistula is described from the proximal Circumflex artery ending in the Superior Vena Cava 3 cm from the SVC-right atrium junction (stable). The maximum diameter of the fistula is 8 mm (stable) (Fig. 3). But there are new findings: significant stenosis of the left anterior descending artery (LAD), the trunk ostium of the diagonals and 2nd diagonal.

**Fig. 4:**
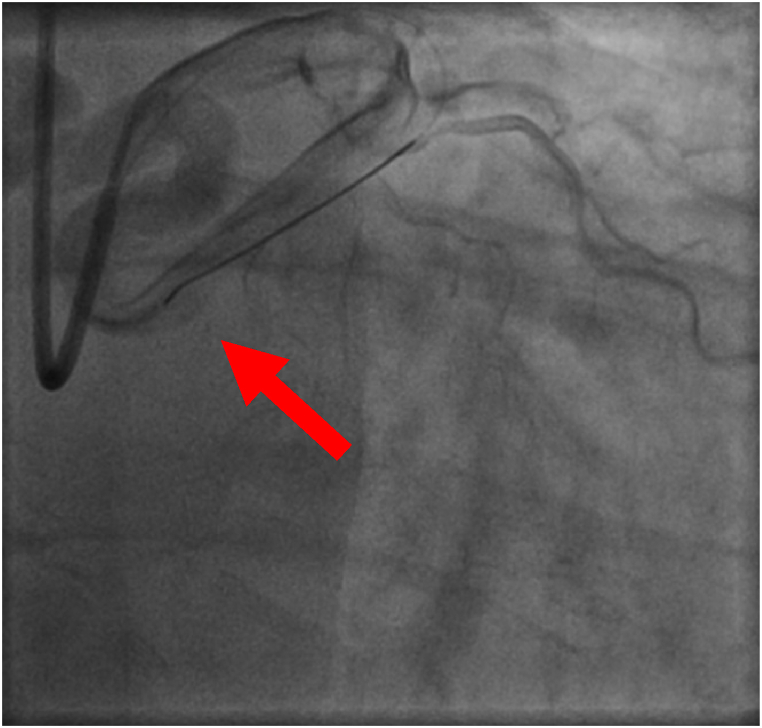
Fistula on coronary angiography.

**Fig. 5:**
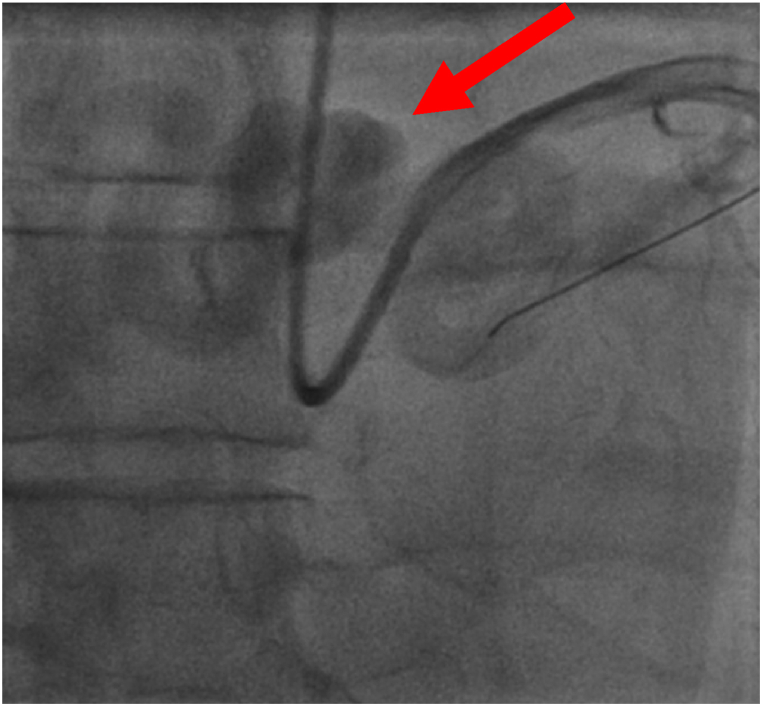
Fistula on coronary angiography.

Therefore, a coronary angiography is performed to confirm the pathological Cardiac CT scan in November 2020 and shows significant LAD1 stenosis and moderate downstream atheroma; vascular theft on the left Circumflex artery, no opacification, voluminous Circumflex fistula heading towards the right atrium; 40 % distal right coronary artery stenosis (Fig. 4, Fig. 5).

**Fig. 6:**
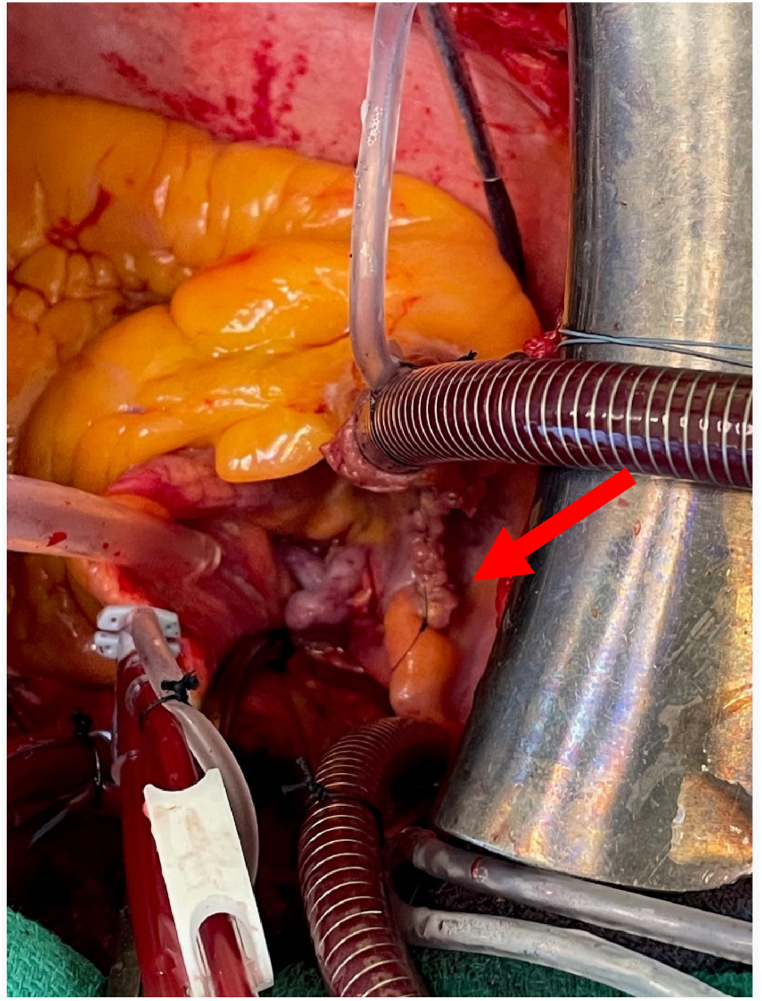
Fistula's ligation surgery.

Because of a coronary artery disease that has become significant and is considered as a multivessel coronary disease but also due to the patient's good general condition and active lifestyle and the giant size of the fistula, cardiac surgery is decided with two aims: aorto-coronary bypass and fistula closure. The procedure is as follows: distal AMIG-IVA bypass and ligation of the coronaro-cava fistula from the Circumflex artery (Fig. 6). Surgery is a success. Subsequently, a minimal right-sided pericardial effusion was noted without clinical impact. The patient then underwent a recovery period in cardiovascular rehabilitation, achieving complete recovery of autonomy and usual physical activity. The echocardiogram at 6 months post-surgery was strictly normal. A stress test was performed one year after the surgery and only showed non-specific inferolateral repolarization abnormalities.

## 3 Discussion

Coronary fistulas, of rare etiology, are not influenced by ethnic origin or gender [1]. The tortuosity depends on the origin and course of the fistula [2]. Fistula's origin from the Circumflex artery is the rarest (18 % versus 30 % for left ventricular artery and 52 % for right coronary artery) [1]. 90 % end up in the right heart, of which 40 % in right ventricular, then come in order of frequency the right atrium, the coronary sinus and the pulmonary trunk [1,2]. The rarest ending (1 % in the literature) concerns the superior vena cava [1]. Variable symptoms found in the literature are angina, murmur, dyspnea, arrhythmia, heart failure, pulmonary hypertension, pneumonia, endocarditis. Other symptoms such as syncope, palpitations, arrhythmias are uncommon [1,2,7]. Concerning paraclinical examinations, the transthoracic echocardiography visualizes fistula's origin, course and termination while Doppler shows fistula blood flow [1]. Coronary angiography ± IVUS, even if more invasive, is anatomical superior and so the gold standard [1,7]. Cardiac catheterization and angiography allow detailed anatomy (size, origin, route, presence of stenosis, drainage site) as well as haemodynamic impact [2]. Coronary CT is a very interesting examination for fistula's diagnosis but also for describing obstructions or coronary steal (“coronary steal phenomenon”) [1,6,7]. As for cardiac MRI, it confirms diagnosis [2] but does little to determine distal path [7]. Management depends on symptomatic character and age at diagnosis [1]. A simple follow-up is recommended in case of small or medium caliber in asymptomatic patients [1,4]. Based on The American College of Cardiology and American Heart Association Guidelines (2008), closure is considered in all other cases and consists of [1,2,5,7]:

- Percutaneous closure: proximal fistula with a single drainage site/distal narrowing of the fistula accessible to dedicated equipment/non-tortuous vessels/severe cardiac comorbidities
- Surgical closure in other cases

In our case, even though the fistula was large, the patient remained asymptomatic for several years, and all annual paraclinical examinations showed no morphological or hemodynamic impact. However, if any anomaly had been found, intervention would have been considered more promptly.

## 4 Conclusion

Coronary fistula is a rare condition often asymptomatic but that can lead to surgical or interventional closure in some cases.

## CRediT authorship contribution statement

**F. Bouriche:** Writing – review & editing, Writing – original draft, Methodology, Investigation, Conceptualization. **S. Yvorra:** Validation, Supervision. **V. Gariboldi:** Visualization, Data curation.

## Data availability statement

Data are available on request. The patient was informed and gave his consent.

## Declaration of competing interest

The authors declare that they have no known competing financial interests or personal relationships that could have appeared to influence the work reported in this paper.
